# HS-SPME Gas Chromatography Approach for Underivatized Acrylamide Determination in Biscuits

**DOI:** 10.3390/foods10092183

**Published:** 2021-09-14

**Authors:** Cláudia P. Passos, Sílvia Petronilho, António F. Serôdio, Andreia C. M. Neto, Dylan Torres, Alisa Rudnitskaya, Cláudia Nunes, Kristína Kukurová, Zuzana Ciesarová, Sílvia M. Rocha, Manuel A. Coimbra

**Affiliations:** 1 Associated Laboratory for Green Chemistry (LAQV-REQUIMTE), Department of Chemistry, University of Aveiro, 3810-193 Aveiro, Portugal; silviapetronilho@ua.pt (S.P.); antonio.serodio@gmail.com (A.F.S.); andreiacat@outlook.pt (A.C.M.N.); dylantorres@ua.pt (D.T.); claudianunes@ua.pt (C.N.); smrocha@ua.pt (S.M.R.); mac@ua.pt (M.A.C.); 2 Chemistry Research Centre-Vila Real, Department of Chemistry, University of Trás-os-Montes and Alto Douro, 5001-801 Vila Real, Portugal; 3 Department of Chemistry, Aveiro Institute of Materials (CICECO), University of Aveiro, 3810-193 Aveiro, Portugal; 4 Centre for Environmental and Marine Studies (CESAM), Department of Chemistry, University of Aveiro, 3810-193 Aveiro, Portugal; alisa.rudnitskaya@gmail.com; 5 National Agricultural and Food Center, Food Research Institute, 824 75 Bratislava, Slovakia; kristina.kukurova@nppc.sk (K.K.); zuzana.ciesarova@nppc.sk (Z.C.)

**Keywords:** acrylamide, HS-SPME, GC-MS, ion extraction, cookies, mitigation

## Abstract

Acrylamide (AA) is a food contaminant in thermally processed products that is object of tight control. A simple and easy-to-apply methodology for routine monitoring of AA levels in food products could allow producers to be players in the control of their own products. In this work, a simple methodology for AA quantification without derivatization was developed for biscuits, for which the benchmark levels recommended by EFSA are 350 µg/kg, and 150 µg/kg for biscuits for infants and young children. Headspace-solid phase microextraction (HS-SPME) was used in 120 mL screwed-cap vials with a carboxen/polydimetylsiloxane fiber, 4 g of biscuits, and 10 mL of water during 15 min at room temperature under stirring. The addition of 30 mL of propanol under stirring during 15 min at room temperature and 15 min at 60 °C was used to promote AA transfer to the headspace. The fiber exposure was 45 min. A gas chromatography-mass spectrometry analysis allowed to obtain an external calibration curve at *m*/*z* 71, with linearity R^2^ > 0.99 and precision RSD < 9%. The detection and quantification limits were 27.4 µg/kg and 91.5 µg/kg, respectively. The methodology was successfully used in biscuits with lower AA amount, where mitigation strategies (asparaginase or pectate) were applied.

## 1. Introduction

Acrylamide (AA) is a food contaminant that can be found in processed products as chips, fries, biscuits, bread, and coffee. It occurs in foods submitted to high temperatures under low moisture conditions, as those used in frying, baking, and roasting [1,2]. The presence of acrylamide in heated foods has been considered an important food-related concern by international authorities since 2002, since this compounds was classified by the International Agency for Research on Cancer (IARC) as a possible human carcinogen (class 2a) since 1994 [3]. To contribute to monitor and regulate its levels in food matrices by producers and authorities [4,5], it is important to have a simple, accessible, and reliable methodology to monitor AA concentration in thermally processed foods. Biscuits, as a major food source of acrylamide exposure for baby and infants [6,7], and a product of high consumption levels among young children [8], is one example of food products that should have a tight control.

Liquid chromatography-tandem mass spectrometry (LC-MS/MS) is the reference method for AA determination in food [9,10,11,12,13,14,15]. This methodology has the advantage of not requiring AA derivatization, but an extensive clean-up methodology for sample defatting and deproteinization is necessary [9]. Due to the complexity of food matrices, which account for a large number of water-soluble compounds that affect chromatographic separation and interfere with detection method, LC requires isotope-labeled internal standards such as d3-acrylamide [16,17,18] and ^13^C-3-acrylamide [16].

The gas chromatography technique can also be used for AA quantification [19,20]. However, an AA derivatization is always required to promote its volatility and thermal stability [9,21]. Furthermore, the direct injection of AA results in a high background noise that limits the possibility to achieve a low detection limit [9] and the possibility to generate in situ AA in the heated GC injection port in the presence of AA precursors (sugars and asparagine) co-extracted from the food matrix [9,22,23]. Some methods proposed in literature are compiled in Table 1. The use of solid-phase microextraction (SPME) in headspace mode (HS) [24,25,26,27] minimizes the purification requirements. Due to AA high-water solubility, it can be easily extracted from food matrices with water at room temperature [23]. However, this AA high-water solubility compromises its transfer to the headspace [25], representing a drawback for its implementation. Consequently, a methodology that allowed the simultaneous extraction and quantification of AA, without derivatization and implementation of complex steps, is a major need.

The aim of this study was to develop a simple and reliable methodology to quantify AA using HS-SPME followed by gas chromatography coupled with quadruple mass spectrometry detection (GC-qMS) in ion extraction chromatography (IEC) mode (*m*/*z* 71 and 55). AA extraction conditions including the type of fiber coating, the extraction temperature and time, and the amount of organic solvent in the aqueous solution were optimized. The applicability of this methodology was further tested in biscuit samples, including the ones with low AA content being produced using mitigation strategies (asparaginase and pectate addition).

## 2. Materials and Methods

### 2.1. Reagents and Materials

Acrylamide (≥98.0%) (AA) was purchased from Sigma-Aldrich (Saint Louis, MO, USA). Sodium chloride (99.5%) and 1-propanol (99.9%) were supplied from VWR chemicals (Lisbon, Portugal). Deionized water was used in the experiments. Two AA individual stock solutions A (5.0 g/L) and B (0.010 mg/L) were prepared in water and stored at 4 °C until use. Thermostatization of stock solutions occurred 30 min before starting each day experiments at room temperature. The screw cap glass vials, headspace-solid phase microextraction (SPME) fibers, and SPME holder for manual sampling were purchased from Supelco (Bellefonte, PA, USA). Prior to the first usage, the fibers were conditioned according to the manufacturer’s recommended procedures.

### 2.2. Biscuit Samples

Wheat-based biscuits were produced in 2020, by a local biscuit manufacturer (DanCake, Portugal) and the list of ingredients used in the recipe is presented in Table S1. To test the applicability of the developed methodology different biscuit samples were analyzed: the original producer recipe, used as reference, and 3 different biscuits produced using modification of the original recipe to test acrylamide (AA) mitigation strategies: (1) replacing 50% of wheat-flour with rice flour; (2) co-addition of 3.5% asparaginase (*w*/*w*, in relation to the flour content); (3) co-addition of 2% pectate (*w*/*w*, in relation to the flour content) [29]. The samples were grinded (ca. 10 g from a total of 160 g), homogenized using a pestle, and immediately analyzed. From each biscuit formulation at least 3 independent aliquots were analyzed.

### 2.3. Fiber Selection for AA Headspace-Solid Phase Microextraction

To evaluate the most appropriate fiber for AA HS-SPME, 6 different fibers with 1 cm length were used, followed by GC with a flame ionization detector (FID): polydimetylsiloxane (PDMS, 100 μm); polyacrylate (PA, 85 μm); carbowax/divinylbenzene (CW/DVB, 65 μm), carboxen/polydimetylsiloxane (CAR/PDMS, 75 μm); polydimetylsiloxane/divinylbenzene (PDMS/DVB, 65 μm); and divinylbenzene/carboxen/polydimetylsiloxane (DVB/CAR/PDMS, 50/30 μm) covering different coating characteristics. Tested fibers had different ranges of coating length (65–100 µm), polarity (nonpolar, polar, bipolar) and extraction mechanism (absorption or adsorption) (Table S2—Supplementary Data) [30].

A 120 mL sealed screw cap glass vial (Schott Duran) containing 2 g of NaCl was used for headspace generation. The aqueous solution was prepared by transferring the solvents in the following order: 19 mL of distilled water, 20 mL of propanol, and 1 mL of AA stock solution A (5.0 g/L) and stirred for 15 min at room temperature (magnetic bar, 40 × 0.9 mm, at 200 rpm). The screw cap glass vial was placed in a thermostatized bath (VWR International-Material de Laboratório, Lda, Alfragide, Portugal) adjusted to 60.0 ± 0.1 °C for 15 min, and then the SPME fiber was inserted in the vial headspace for 45 min, in a total of 90 min. All measurements were performed at least in triplicate, each one corresponding to one independent aliquot.

### 2.4. Optimization of HS-SPME Extraction Conditions

Optimization of HS-SPME extraction conditions was carried out using Box-Behnken experimental plan with 3 experimental factors: temperature (T), propanol volume (V), and fiber exposure time (t), at 3 levels, in a total of 39 experiments (13 conditions, 3 replicates each) (Table 2). Acrylamide chromatographic peak area was used as response variable.

Experimental factor levels were selected according to the HS-SPME specifications. Minimum and maximum temperatures were respectively set to warranty no dependence on laboratory temperature conditions (≥30 °C) and avoid sample deterioration and formation of contaminants or artifacts (≤60 °C) [31]. The total solution volume with different water to propanol ratios was always maintained at 40 mL in a 120 mL crew cap vial corresponding to a ratio of the volume of the liquid phase to the headspace volume (1/β) of 0.5. The HS-SPME procedure, that included thermostatization and fiber exposure, was always 60 min.

The HS-SPME conditions were initially determined using a GC with a flame ionization detector (FID) with a high AA concentration, stock solution A (5 g/L). To approach the range of AA known to exist in biscuits, the amount used in the following trials was reduced using AA stock solution B (0.01 g/L) and using a GC-qMS for quantification. Maintaining HS-SPME temperature at 60 °C, and thermostatization and fiber exposure times of 15 and 45 min respectively, a full factorial design for the propanol (V) volume factor was performed using 0, 10, 20, 30, and 40 mL of propanol for a total volume of 40 mL, assuming additive volumes. The limiting conditions, namely 0 and 40 mL of propanol, previously excluded from the Box-Behnken design were also included in this new data set. The glass vial was placed in a thermostatized bath adjusted to 60 °C ± 0.1°C for 15 min, and then the CAR/PDMS fiber (selected fiber) was inserted in the headspace for 45 min. All measurements were performed at least in three independent replicates.

### 2.5. Testing the Methodology in Biscuits

**Figure 1:**
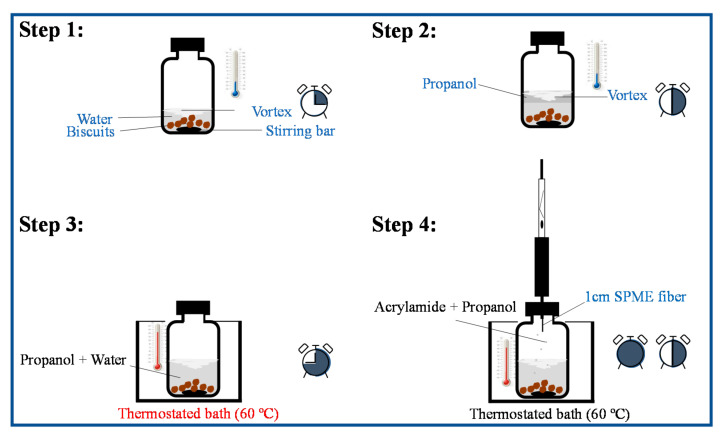
Schematic representation for the HS-SPME procedure applied to extract AA from wheat biscuits. The HS-SPME procedure includes 4 steps: 1—AA water extraction from the solid matrix, 15 min); 2—addition of propanol to enhance AA volatilization to the vial headspace (+15 min); 3—thermostatization of the mixture (+15 min); 4—SPME fiber exposure to the vial headspace (+45 min). The clock represents the time evolution along the 4 steps sequence (total of 90 min) is included.

Manual grinding using a porcelain mortar and pestle (LaborXing, 13 × 6.5 cm of mortar diameter and height, respectively and 9.7 cm of pestle length) followed by dispersion in water was used to reduce the heterogeneity of 4 g of wheat biscuit sample aliquots. At least three aliquots from the same biscuit formulation were used. The milled biscuits were added to the 120 mL screw cap glass vial containing a stirring bar (40 × 0.9 mm, 200 rpm). For AA extraction, 10 mL of water were added. The screw cap glass vial was closed and the suspension was stirred for 15 min at room temperature to soak and swallow the solid matrix and facilitate AA extraction [16] (Figure 1, step 1). To the same screw cap vial, 30 mL of propanol were added, in accordance with the optimized extraction conditions (Section 2.4). The closed vial was stirred for 15 min at room temperature (Figure 1, step 2). Then, the glass vial was placed in a thermostated bath adjusted to 60 °C ± 0.1 °C for 15 min (Figure 1, step 3), and the CAR/PDMS fiber was inserted in the headspace for 45 min (Figure 1, step 4). All measurements were performed at least for 3 independent replicates and blanks between 3 replicates were performed.

### 2.6. GC-FID Conditions

For the selection of the best fiber in Section 2.3 and HS-SPME optimization in Section 2.4, the HS-SPME fiber was manually introduced into a GC injection port at 250 °C (equipped with a glass liner, 0.75 mm I.D.) and kept for 3 min for desorption. The desorbed volatile compounds were separated in a PerkinElmer Clarus 400 gas chromatographer equipped with a DB-FFAP column (30 m × 0.32 mm I.D. × 0.25 μm film thickness) supplied by Agilent J&W Scientific (Folsom, CA, USA) connected to a flame ionization detector. Hydrogen (Air Liquid, Portugal) was used as the carrier gas. The injections were performed in splitless mode. The GC oven temperature was programmed as follows: held at 60 °C for 1 min then ramped at 20 °C/min to 120 °C, followed by a second ramp at 2 °C/min to 140°C, and a third ramp at 20 °C/min to 250 °C, and held there for 2 min. To avoid any cross-over contamination due to own fiber coating or sample type (AA solutions or biscuits), blanks were run between sets of three analyses, corresponding to the analysis of the fiber coating not submitted to any extraction procedure.

### 2.7. GC-qMS Conditions

For AA detection in biscuits the HS-SPME fiber with a CAR/PDMS coating was manually introduced into the GC injection port at 250 °C (equipped with a glass liner, 0.75 mm I.D.) and kept for 3 min for desorption. The desorbed volatile compounds were separated in an Agilent Technologies 6890N Network gas chromatographer equipped with a DB-FFAP column (60 m × 0.25 mm I.D. × 0.25 μm film thickness) supplied by Agilent J&W Scientific (Folsom, CA, USA) connected to an Agilent 5973N quadrupole mass selective detector. Helium (Air Liquid, Portugal) was used as the carrier gas at a flow rate of 1.7 mL/min. The injection was performed in splitless mode. The GC oven program temperature was the same as described in Section 2.6. For the MS system, the temperatures of the transfer line, quadrupole and ionization source were 250 °C, 150 °C, and 250 °C, respectively. Electron impact mass spectra were recorded at 70 eV and the ionization current was about 30 μA. A delay time of 7 min was used. Under these chromatographic conditions, the run was 18 min. The acquisition was performed in an ion extraction chromatography (IEC) mode (71 and 55 *m*/*z*, for AA monitoring, with higher proportion for 71).

### 2.8. Method Validation

Reproducibility was expressed as relative standard deviation (RSD). Signal acquisition and data processing were performed using the HP ChemStation (Agilent Technologies, Santa Clara, CA, USA). Acrylamide calibration curve was built within volumes of 1.6 and 20.4 µg/vial. The concentration levels per vial (µg/vial) were converted into µg/kg of sample, taking the total volume of 40 mL and 4 g of biscuits into account. The limits of detection (LoD) and quantification (LoQ) were defined as the lowest estimated AA concentration (µg/kg) greater than 3 and 10 times of the noise levels (S/N > 3 and 10), respectively [32].

For method validation, several commercial wheat biscuits, using at least 3 different samples from the same formulation, were assessed by HPLC. For the biscuits with asparaginase it was requested an external analysis from a certified laboratory.

### 2.9. Statistical Analysis

Analysis of effects was conducted using ANOVA model comprising all main effects and their interactions. All calculations were made in Unscramble 9.7. The significance of the effects in ANOVA model was tested at the 95% probability level.

## 3. Results and Discussion

To develop a simple and reliable methodology for acrylamide (AA) quantification, extraction conditions including the SPME fiber coating, extraction temperature and time, and amount of organic solvent in the aqueous solution were optimized. The applicability of HS-SPME methodology was validated in biscuit samples.

### 3.1. Selection of SPME Fiber for Acrylamide Headspace Extraction

**Figure 2:**
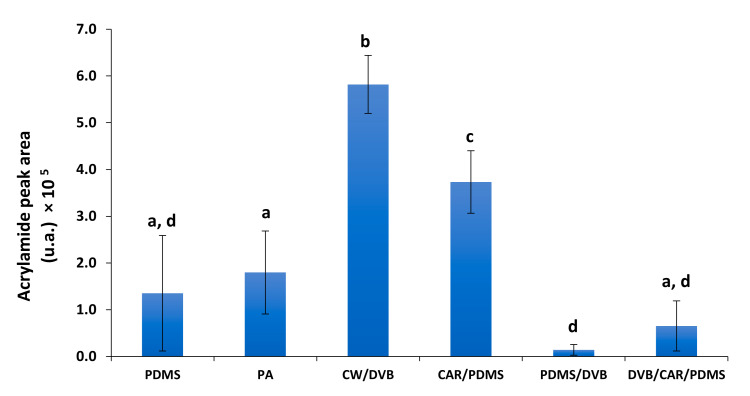
AA peak area (expressed as arbitrary units) obtained as function of different tested fibers. PDMS—polydimetylsiloxane; PA—polyacrylate; CAR—carboxen; DVB—divinylbenzene; CW—carbowax. Different letters represent values that are significantly different (*p* < 0.05).

A total of 6 SPME fibers coating were tested: PDMS, PA, CW/DVB, CAR/PDMS, PDMS/DVB, and DVB/CAR/PDMS, covering different coating characteristics (Table S2). Since several GC-FID analyses were performed, an example of a chromatogram obtained for a concentration of 5 mg/vial was included in Figure S1. The best result for AA extraction was obtained for CW/DVB fiber (Figure 2).

PDMS/DVB and DVB/CAR/PDMS fibers showed the worst results, although they also contain a DVB coating. This coating polymer has been described to be more suitable for medium- and high-molecular weight analytes [33,34], which may explain the inefficiency for AA, a low-molecular weight compound. PDMS coating is recommended for non-polar compounds [34], which explains its low capacity towards AA. The PDMS/DVB has been used for AA extraction using HS-SPME, but only after AA silylation [25]. The best results were obtained using CAR/PDMS and CW/DVB fibers. These two fiber coatings are bipolar and adsorption-type (Table S2). CAR is a porous carbon polymer with micro-, meso-, and macropores of 6–50 Å. When blended with PDMS [35,36], it has been suggested for low molecular weight (C2–C12) compounds extraction [33,34,35,37] with good results for high polarity ones, including the Maillard intermediates [38]. Therefore, the 75 µm CAR/PDMS fiber coating thickness recommended for the compounds in the molecular weight range of 30–225 Da [37], was considered suitable for AA (71 Da). Indeed, CAR/PDMS fiber exhibited higher extraction efficiency (*p* < 0.05) compared to other fibers, although slightly lower than CW/DVB. Similar results were observed when using these fibers for direct immersion sampling [24]. Although CW/DVB fiber showed better results than CAR/PDMS (*p* < 0.05), as CW/DVB fiber is no longer available in the market, CAR/PDMS fiber was chosen as the best option for AA extraction using HS-SPME. The use of a polar fiber, such as PA was also considered. However, the liquid polyacrylate polymeric coating was degraded by propanol, the solvent used to promote the transfer of AA to the vial headspace. Based on all these observations, the optimization of SPME extraction conditions were performed for CAR/PDMS fiber.

### 3.2. Optimization of SPME Extraction Conditions

HS-SPME conditions using CAR/PDMS fiber were optimized by evaluating the effect of three factors on AA extraction: temperature (T), propanol volume (V), and fiber exposure time (t). The experimental plan with 13 experimental runs generated using a Box–Behnken design is shown in the Table 2. The results of analysis of variance of the designed experiments were obtained using ANOVA model comprising all main effects and their interactions (Table 3).

According to ANOVA, only the main effects from the propanol volume (X_V_) and the fiber exposure time (Xt) were statistically significant (*p* < 0.05), while no interaction between factors (X_T_X_V_, X_T_X_t_, and X_V_X_t_) was found to be significant. Temperature (X_T_) did not present a significant impact on the extraction conditions (*p* > 0.05, Table 3).

**Figure 3:**
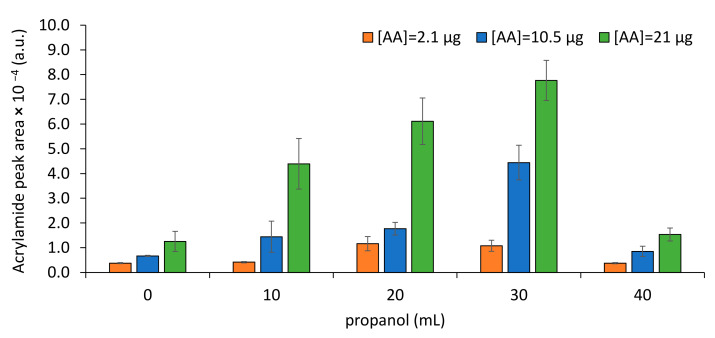
Impact of propanol addition towards AA extraction to SPME fiber in a total of 40 mL of solution.

Under the tested conditions, the propanol volume (V) is the most significant variable. When using only water, the chromatographic areas were low (<1.5 × 10^4^, Figure 3). However, higher AA extractability was observed when higher proportions of propanol were used (lowest *p*-value, negative *b*-coefficient). Nevertheless, propanol alone resulted in low GC chromatographic areas. Due to AA high solubility in water (390 g/L), its extractability to the headspace is low, explaining the low AA peak areas determined when only water is used (Figure 3). Propanol is immiscible in water forming two phases in the presence of salt [39]. Similar to a solubility modifier effect, the saturation of water with propanol seems to reduce AA solubility, favoring its volatilization to the headspace. Propanol has already been used to AA extraction from food samples [22,28]. Nevertheless, because AA solubility in propanol is also high [22,40], when using only propanol, low GC chromatographic areas were observed. Mixture of propanol with water seems to decrease the solubility of AA, resulting in higher AA concentration in the headspace than when these solvents were assayed alone. In the studied samples, water is a solvent serving for AA extraction from solid matrix, while propanol is a solvent promoting AA volatilization in the presence of water.

**Figure 4:**
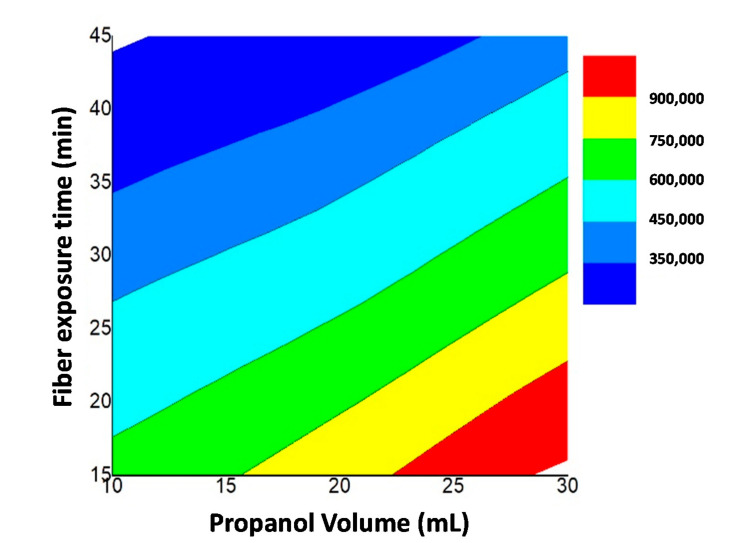
Response surface for the effects of the fiber exposure time (X_t_) and propanol volume (X_V_) at a constant temperature of 60 °C on AA extraction (peak area).

In the case of the fiber exposure time (t) factor, higher AA extractability was achieved when using 15 min of thermostatization followed by 45 min of fiber exposure to the analyte (negative *b*-coefficient). According to Figure 4, higher AA extractability was obtained when using the combination of time and solvent conditions: 10 mL of water and 30 mL of propanol thermostatized during 15 min at 60 °C followed by a fiber exposure of 45 min.

### 3.3. Validation of the HS-SPME/GC-MS Methodology for Acrylamide Quantification

**Figure 5:**
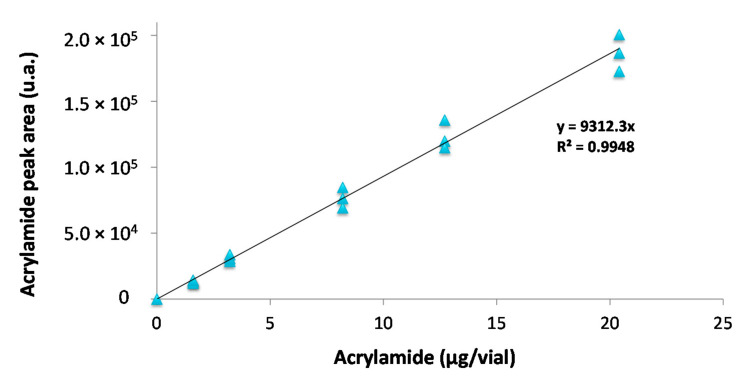
Acrylamide external calibration curve (RSD < 8.9%).

An external calibration curve was built using increments of AA in model aqueous solutions (Figure 5). Representative chromatograms (standard solution and biscuits samples) and respective mass spectrum for AA were included as Supplementary Materials (Supplementary Figure S2).

A good linearity was obtained with a coefficient of determination (R^2^ > 0.99). Furthermore, the developed methodology showed high precision (RSD < 9%), with limits of detection, LoD = 0.11 µg/vial, and quantification, LoQ = 0.37 µg/vial. Considering that 4 g of biscuit in a 120 mL SPME vial are used for sample preparation, the LoD and LoQ can be estimated to be 27.4 µg/kg_biscuits_ and 91.5 µg/kg_biscuits_, respectively. Contrarily to the methods described in bibliography (Table 1) this work proposes a methodology that does not requires clean-up nor derivatization of acrylamide. Because the LoQ is still below the benchmark level for AA in biscuits (350 µg/kg_biscuits_) recommended by the European Food Safety Agency [5], the proposed methodology could be implemented in routine analysis for AA monitoring in biscuits.

### 3.4. Acrylamide Quantification in Biscuits

**Figure 6:**
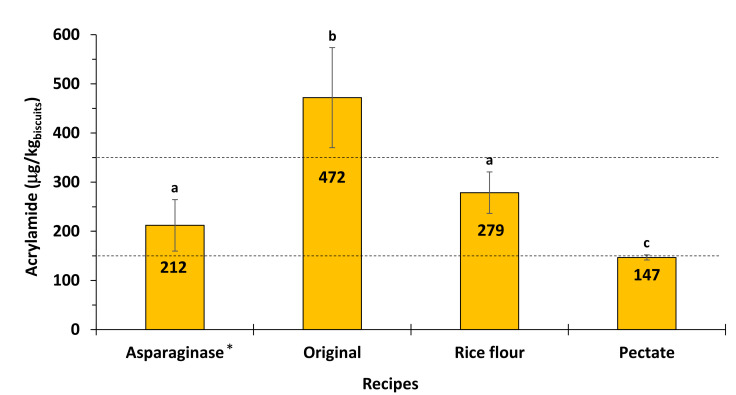
Acrylamide (AA) content determined in wheat meal biscuits. Different letters represent values that are significantly different (*p* < 0.05). Dashed lines represent the 350 µg/kg benchmark level for the presence of AA in biscuits and the 150 µg/kg for the benchmark level for the presence of AA in biscuits for infant and young children. * AA biscuit sample also quantified by an external certified laboratory using a conventional HPLC method.

The developed methodology for AA quantification was tested in a commercial wheat biscuit, treated with asparaginase. The biscuits contained 203 ± 45 µg/kg of AA, which was quantified by in an external certified laboratory using a conventional HPLC method. Using the developed methodology, the content of AA in these biscuits treated with asparaginase was found to be 212 ± 34 µg/kg, which is in accordance with the values provided by the certified laboratory (Figure 6).

Biscuits prepared using original wheat recipe contained higher amount of AA, 472 µg AA per kg of biscuits compared to the biscuits prepared using mitigation methodologies (Figure 6). The mitigation effect of the addition of asparaginase [41] is due to the conversion of free asparagine into aspartic acid, which is no longer able to form AA [42]. According to the obtained results, addition of asparaginase to the wheat flour biscuits allowed to decrease AA content below the benchmark level recommended by EFSA for biscuits (350 µg/kg) [5], demonstrating the relevance of asparaginase as a mitigation strategy in biscuits.

Substitution of 50% of the wheat flour by the same amount of rice flour in biscuit recipe also resulted in the decrease of AA levels, to 279 µg per kg of biscuits, value below the recommended benchmark value [5]. Effect of rice flour addition is due to the lower contents of free asparagine in this flour compared to wheat [43].

The most effective mitigation methodology was found to be addition of 2% of pectate to biscuit dough allowing AA content to decrease to 147 µg per kg of biscuits (Figure 6). These results are in accordance with AA mitigation strategy by pH lowering of the dough using acidic polysaccharides [29].

## 4. Conclusions

In this work, we propose a simple methodology for AA quantification based on direct extraction followed by quantification by GC-qMS (HS-SPME/GC-qMS) that does not require a complex sample preparation step.

The biscuits were grinded and suspended in water for AA extraction from the solid matrix. Addition of propanol to the slurry promoted the AA migration to the headspace facilitating its adsorption on the SPME fiber. GC-qMS in ion extraction mode allowed to achieve a limit of quantification of 91.5 µg/kg of biscuits, which is below the 150 µg/kg_biscuits_ benchmark level for AA in biscuits for infants and young children. This sequential analysis, comprising extraction and an agent that modifies acrylamide solubility in water, always using the same vial, can be combined with GC-MS, an equipment that is nowadays available in food industries, allowing a simple and quick quality control by producers.

## Figures and Tables

**Table 1:** Literature survey on methods used to extract and quantify acrylamide in food products.

Extraction Method	Detection Method	Clean Up	AA Derivatization	LoD/LoQ	Food Product	Reference
SLE	LC-HRMS	Yes	No	2.65/5 ppb	Several foods *	[10]
DLLME	UPLC-MS/MS	Yes	No	0.9/3.0 μg/L	Brewed coffee	[11]
SLE	LC-MS/MS	Yes	No	0.3/1 µg/kg	Bread	[13]
SLE	LC-QqQ-MS/MS	Yes	No	LoQ:1.3–1.4 mg/kg	Insect-based products **	[14]
SLE	LC-MS/MS	Yes	No	0.62/1.89 µg/kg	Corn snacks	[15]
SLE	LC-MS	Yes	Yes	6.6/19.6 µg/kg	Potato chips, crispbread, butter, and cookies	[16]
SLE	GC-MS	Yes	No	4.0/11.8 µg/kg		[16]
SLE	GC-MS	Yes	No	5.6/16.7 µg/kg		[16,28]
SLE	LC-MS/MS	Yes	No	9.2/12.5 µg/kg	Chocolate powder, roasted and soluble coffee	[17]
SPE	LC-MS	Yes	No	3.55/11.8 µg/kg	Biscuits	[18]
DSPE	GC-MS	Yes	No	6.7/20.3 µg/kg	Dark Chocolate	[19]
DLLME	GC-MS	Yes	No	0.6/2 µg/kg	Roasted nuts and seeds	[20]
HS-SPME	GC-MS	No	Yes ***	1/3 µg/kg	Coffee Beans	[21]
DSPE	GC-MS	Yes	No	LoQ:15–40 µg/kg	Food products	[22]

SLE: Solid-Liquid Extraction; DLLME: Dispersive Liquid–Liquid MicroExtraction; SPE: Solid-Phase Extraction; DSPE: Dispersive Solid-Phase Extraction; HS-SPME: Headspace-Solid Phase Microextraction; LC-HRMS: Liquid Chromatography with High Resolution Mass Spectrometry; UPLS-MS/MS: Ultra-Performance Liquid Chromatography tandem Mass Spectrometry; LC-MS/MS: Liquid Chromatography-tandem Mass Spectrometry; HPLC-QqQ-MS/MS: High-Performance Liquid Chromatography coupled to Triple Quadrupole Mass Spectrometry; GC-MS: Gas Chromatography–Mass Spectrometry; * This methodology was applied in several acrylamide containing foods: cookies, French fries, ground coffee and brewed coffee. ** bars, crackers, and flours. *** Silylation.

**Table 2:** Experimental plan using the Box-Behnken design for the three variables under study: temperature (*T*), propanol volume (*V*), and fiber exposure time (*t*).

	Variables		
Condition	*T* (°C)	*V* (mL)	*T* (min)
1	40	30	30
2	60	30	30
3	40	10	30
4	60	10	30
5	40	20	45
6	60	20	45
7	40	20	15
8	60	20	15
9	50	30	45
10	50	10	45
11	50	30	15
12	50	10	15
13	50	20	30

Operating conditions are temperature (*T*, °C), propanol volume (*V*, mL), and fiber exposure time (*t*, min).

**Table 3:** Sources of variation in the ANOVA models for acrylamide extraction (peak area).

Variable	*b*-Coefficient	*p*-Value
Model	-	**0.016**
Intercept	516,900	
X_T_	6299	0.442
X_V_	−467,400	**0.007**
X_t_	−411,900	**0.016**
X_T_X_V_	−83,380	0.257
X_T_X_t_	42,320	0.562
X_R_X_t_	88,900	0.227

The notation “-“ represents no *b*-coefficient. Bold numbers refer to significant variation sources (*p* < 0.05) for both model and parameters. The operating conditions are temperature (T, ºC), propanol volume (V, mL), and the fiber exposure time (t, min). X_T_—effect of temperature, X_V_—effect of propanol volume, X_t_—effect of fiber exposure time, X_T_X_V_—interaction of the effects of temperature and propanol volume, X_T_X_t_—effect of the interaction of temperature and fiber exposure time, and X_V_X_t_—effect of the interaction of propanol volume and fiber exposure time.

## Supplementary Materials

The following are available online at https://www.mdpi.com/article/10.3390/foods10092183/s1, Table S1: List of ingredients used in the biscuit recipe, Table S2: Types of commercially available fiber coatings used in this work, Figure S1: GC-FID chromatogram with acrylamide with a concentration of 5 mg/vial obtained when using a PA fiber. Figure S2. (a) GC-MS chromatograms: (left) standard AA solution (6.4 µg/vial) and (right) biscuits sample with acrylamide detection (9.31–9.30 min); (b) spectrum for AA using the ion extraction chromatography (IEC) mode (71 and 55 *m*/*z* monitoring) and (c) literature full scan spectrum data for propenamide (AA).
