# Supplementary material for: HS-SPME Gas Chromatography Approach for Underivatized Acrylamide Determination in Biscuits
Source: Foods. 2021 Sep 14;10(9):2183. doi: 10.3390/foods10092183 (PMC8470632; doi:10.3390/foods10092183)
Supplement: Supplementary file 1 [file foods-10-02183-s001.zip › foods-1350617-supplementary.pdf]

Supplementary data

HS-SPME gas chromatography approach for underivatized acrylamide determination in biscuits

Cláudia P. Passos*, Sílvia Petronilho, António F. Serôdio, Andreia C.M. Neto, Dylan Torres, Alisa Rudnitskaya, Cláudia Nunes, Kristína Kukurová, Zuzana Ciesarová, Sílvia M. Rocha and Manuel A. Coimbra

- Correspondence: cpassos@ua.pt; Tel.: + 351 234 370706

Table of Contents

Table S1. List of ingredients used in the biscuit recipes. 3

Table S2. Types of commercially available fibre coatings used in this work 3

Figure S1. Example of a GC-FID chromatogram obtained for AA with a concentration of 5 mg/vial using a PA fibre. 4

Figure S2. GC-MS chromatograms obtained for (a): (left) standard AA solution (8 µg/vial) and (right) biscuits sample with AA detection (retention time: 9.31 min); ion extraction spectrum of AA using *m/z* 71 and 55 (b), and literature full scan spectrum data for AA (c). 5

Table S1. List of ingredients used in the biscuit recipes.

	Modifications		
**Original**	**Recipe 1**	**Recipe 2**	**Recipe 3**
Cereals (68%): wheat flour, rice flour, whole wheat flour, rye flour	✓	Substitution of 50% of the wheat flour content rice flour	✓
Sunflower oil	✓	✓	✓
Sugar	✓	✓	✓
Glucose-frutose syrup	✓	✓	✓
Salt	✓	✓	✓
Raising agents (Sodium, Calcium and Ammonium carbonates)	✓	✓	✓
Vitamins	✓	✓	✓
Skimmed milk	✓	✓	✓
-	+ 3.5% asparaginase*	-	+ 2% pectate*

* w/w, in relation to the total flour content.

Table S2. Types of commercially available fibre coatings used in this work [36].

Stationary phase	Coating (µm)	Polarity	Extraction mechanism
**PDMS**	100	Nonpolar	Absorption
**PA**	85	Polar	Absorption
**CW/DVB***	65	Polar	Adsorption
**CAR/PDMS**	75	Bipolar	Adsorption
**PDMS/DVB**	65	Bipolar	Adsorption
**DVB/CAR/PDMS****	50/30	Bipolar	Adsorption

PDMS- polydimetylsiloxane; PA- polyacrylate; CW- Carbowax; CAR – carboxen; DVB – divinylbenzene. * This fibre has been taken out from the market. ** This stationary phase compromises a 50/30 DVB/CAR on PDMS fibre.

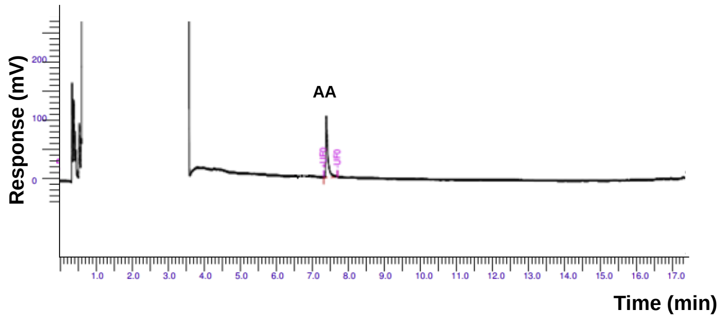

Figure S1. Example of a GC-FID chromatogram obtained for AA with a concentration of 5 mg/vial using a PA fibre.

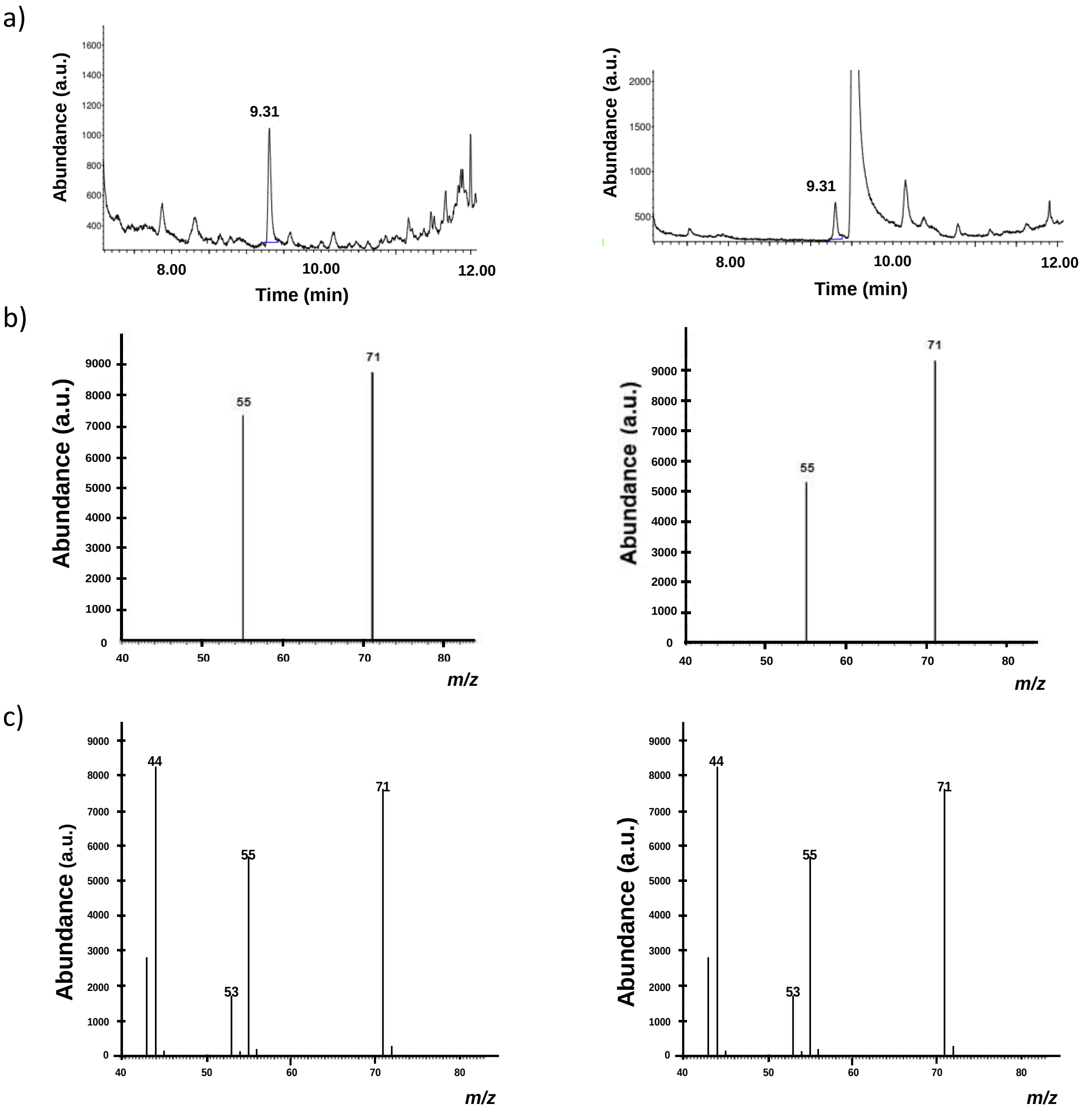

Figure S2. GC-MS chromatograms obtained for (a): (**left**) standard AA solution (8 µg/vial) and (**right**) biscuits sample with AA detection (retention time: 9.31 min); ion extraction spectrum of AA using *m/z* 71 and 55 (b), and literature full scan spectrum data for AA (c).
